# Systemic Vulnerability, as Expressed by I-CAM and MMP-9 at Presentation, Predicts One Year Outcomes in Patients with Acute Myocardial Infarction—Insights from the VIP Clinical Study

**DOI:** 10.3390/jcm10153435

**Published:** 2021-07-31

**Authors:** Diana Opincariu, Ioana Rodean, Nora Rat, Roxana Hodas, Imre Benedek, Theodora Benedek

**Affiliations:** 1 Department of Cardiology, University of Medicine, Pharmacy, Sciences and Technology “George Emil Palade”, 540142 Târgu Mureș, Romania; ioana.rodean@umfst.ro (I.R.); nora.rat@umfst.ro (N.R.); imre.benedek@umfst.ro (I.B.); theodora.benedek@umfst.ro (T.B.); 2 Cardiomed Medical Center, 22 December 1989 Street, No. 76, 540124 Târgu Mureș, Romania

**Keywords:** acute myocardial infarction, predictors for 1 year-MACE, serum inflammatory biomarkers, MMP-9, angiographical characteristics

## Abstract

(1) Background: The prediction of recurrent events after acute myocardial infarction (AMI) does not sufficiently integrate systemic inflammation, coronary morphology or ventricular function in prediction algorithms. We aimed to evaluate the accuracy of inflammatory biomarkers, in association with angiographical and echocardiographic parameters, in predicting 1-year MACE after revascularized AMI. (2) Methods: This is an extension of a biomarker sub-study of the VIP trial (NCT03606330), in which 225 AMI patients underwent analysis of systemic vulnerability and were followed for 1 year. Hs-CRP, MMP-9, IL-6, I-CAM, V-CAM and E-selectin were determined at 1 h after revascularization. The primary end-point was the 1-year MACE rate. (3) Results: The MACE rate was 24.8% (*n* = 56). There were no significant differences between groups in regard to IL-6, V-CAM and E-selectin. The following inflammatory markers were significantly higher in MACE patients: hs-CRP (11.1 ± 13.8 vs. 5.1 ± 4.4 mg/L, *p* = 0.03), I-CAM (452 ± 283 vs. 220.5 ± 104.6, *p* = 0.0003) and MMP-9 (2255 ± 1226 vs. 1099 ± 706.1 ng/mL *p* = 0.0001). The most powerful predictor for MACE was MMP-9 of >1155 ng/mL (AUC-0.786, *p* < 0.001) even after adjustments for diabetes, LVEF, acute phase complications and other inflammatory biomarkers. For STEMI, the most powerful predictors for MACE included I-CAM > 239.7 ng/mL, V-CAM > 877.9 ng/mL and MMP-9 > 1393 ng/mL. (4) Conclusions: High levels of I-CAM and MMP-9 were the most powerful predictors for recurrent events after AMI for the overall study population. For STEMI subjects, the most important predictors included increased levels of I-CAM, V-CAM and MMP-9, while none of the analyzed parameters had proven to be predictive. Inflammatory biomarkers assayed during the acute phase of AMI presented a more powerful predictive capacity for MACE than the LVEF.

## 1. Introduction

Cardiovascular disease (CVD) still remains one of the main causes of death worldwide, and 85% of deaths are caused by acute cerebrovascular and coronary events, especially acute myocardial infarction (AMI) [1]. Despite timely reperfusion therapies, mortality and morbidity in AMI still remains high, as up to 7% and 22%, respectively, at one year after the acute event. Continuous improvement and the identification of new predictive markers for short and long term adverse events following AMI is, therefore, imperative [2]. Over the last decade, several risk prediction models have been developed that take into account the clinical, biological and morphological characteristics of patients presenting with acute coronary syndromes (ACS) [3,4,5,6].

Systemic inflammation holds a major role in plaque vulnerability and rupture, and it also contributes to left ventricular remodeling consequent to AMI [7,8]. Inflammation is fundamental in all pathophysiological processes that lead to the development of atherosclerosis, plaque destabilization and myocardial healing, scarring and remodeling. Furthermore, a large body of data has shown the prognostic utility of inflammatory markers in patients with coronary artery disease (CAD). The concept of “vulnerable patient” was developed almost two decades ago, to define patients that are prone to developing ACS and sudden cardiac death, centered on vulnerable plaques, blood and myocardium. Vulnerable blood, with increased thrombogenicity, is characterized by an increased inflammatory state and an abnormal lipidic profile. It includes increased levels of specific markers for immune activation triggered by various stimuli, from infections to autoimmune disorders, or just by the mere presence of cardiovascular risk factors [9,10].

Several serological biomarkers indicative of inflammation have been studied in relation to atherosclerosis, plaque destabilization, and the occurrence or prognosis of ACS.

C-reactive protein, an acute phase inflammatory reactant, has been proven as a marker and promoter of atherosclerosis [11,12,13,14]. Highly sensitive C-reactive protein (hs-CRP) is a better indicator of cardiovascular risk compared to traditional assays and has been associated with the severity of CAD, plaque vulnerability and future cardio- and cerebrovascular events in patients that have suffered an ACS [15,16,17]. In addition to providing quantitative information on systemic inflammation, hs-CRP promotes endothelial dysfunction by stimulating the expression of adhesion molecules, leukocyte activation, lipid accumulation, platelet aggregation and thrombosis [18]. Interleukin-6 (IL-6), another acute phase inflammatory reactant, promotes leukocyte activation, and has been associated with increased mortality and morbidity in patients with AMI [19,20]. Matrix metalloproteinases (MMP), and especially MMP-9, are responsible for degradation of the extracellular matrix of coronary plaques, which may lead to fatal ACS. In addition, MMPs are implicated in left ventricular remodeling in various forms of cardiomyopathies and post-MI. MMPs enable immune cells and inflammatory mediators to travel within tissues, thus fast-tracking the process of plaque destabilization [21,22]. Recent reports have shown that MMP-9 may hold prognostic value in patients with AMI, and has been associated with long-term adverse events [23,24]. Cell adhesion molecules (CAM), such as intercellular and vascular adhesion molecules (ICAM-1, VCAM-1), as well as selectins, mediate plaque inflammation by stimulating the transmigration of immune cells across the endothelium, under the influence of increased oxidative and mechanical shear stress, ultimately contributing to plaque rupture and thrombosis. Circulating soluble forms of CAMs offer prognostic information in healthy populations and in variate clinical presentations of ischemic heart disease. This is also sustained by significantly increased serum levels of CAMs in patients with ACS compared to stable CAD, and by their correlation with increased levels of cardiac troponins and CRP [20,25,26,27,28].

Systemic vulnerability has a defined role in patient prognosis, although there are several morphological characteristics of AMI patients that can alter short- and long-term outcomes. Such characteristics include the presence of multivessel CAD, the location of atherosclerotic lesions within the coronary tree, the left ventricular function after the acute event, and the presence or absence of acute phase complications (electrical, hemodynamic or mechanical complications) [29,30,31,32].

Several risk prediction scores have been proven to hold a strong predictive capacity for short and long term prognosis. The most widely used TIMI (Thrombolysis in Myocardial Infarction) [33] and GRACE (Global Registry of Acute Coronary Events) [34] scores do not consider systemic inflammatory response, nor coronary morphology nor ventricular function. An integration of biological markers indicative of systemic inflammation with morphological characteristics that encompass the severity of CAD and left ventricular function could improve the prediction of the residual risk of MACE in patients that have suffered an AMI.

Therefore, the aim of this study was to demonstrate the predictive capacity of inflammatory serum biomarkers (hs-CRP, MMP-9, IL-6, adhesion molecules) in association with angiographical parameters characterizing the severity of CAD and LV function, in predicting MACE over the course of a one-year follow-up period, in patients with revascularized AMI (STEMI and NSTEMI).

## 2. Materials and Methods

### 2.1. Patient Selection

This is a prospective follow-up study conducted in the Clinic of Cardiology of the Emergency Clinical County Hospital of Târgu Mureș, Romania, which included 225 consecutive patients with ST segment elevation or non-ST elevation acute myocardial infarction (STEMI and NSTEMI). The diagnosis of AMI was established according to the Fourth Universal Definition of Myocardial infarction [35]. All STEMI patients underwent primary PCI, without previous thrombolytic therapy, under less than 12 h from the onset of symptoms. NSTEMI patients underwent coronary angiography and revascularization of culprit lesions by using the immediate invasive and early invasive strategies, according to the European Guidelines for the management of patients with non-ST elevation acute coronary syndromes. All included patients were administered optimal medical therapy according to the European guidelines in effect, and had received dual antiplatelet therapy in loading doses, Aspirin and a P2Y12 inhibitor (Clopidogrel or Ticagrelor) prior to invasive management.

Patients who had deceased during hospitalization, those who had undergone thrombolysis (in case of STEMI), patients with autoimmune disorders, anti-inflammatory or immunomodulatory treatments 3 months prior to enrollment, those with acute infectious disease (pulmonary, urinary, other) during hospitalization for an index event, with myocarditis, MINOCA (myocardial infarction with non-obstructive coronary arteries), pericarditis, pregnancy or lactation and women of reproductive age who were not using any contraceptive method, allergy and history of allergic reactions to iodine contrast media, severe renal insufficiency (estimated glomerular filtration rate of <29 mm/min/1.73 m^2^), active malignancy or malignancy within the last 1 year prior to enrollment, were excluded.

All patients consented to the use of their clinical data for research, and the study protocol was reviewed and approved by the Ethics Committee of the Hospital and of the University of Medicine, Pharmacy, Sciences and Technology of Târgu Mureș (no. 347/13.12.2017). All study procedures were conducted according to the ethical guidelines of the Declaration of Helsinki.

### 2.2. Study Protocol

All included patients provided information regarding their medical history, previous medications, cardiovascular risk factors, and had undergone a complete clinical examination, 12-lead ECG evaluation, blood sampling for general laboratory testing (complete blood cell count, biochemical testing, myocardial necrosis enzymes) and invasive coronary angiography with revascularization of the culprit lesion, as well as associated coronary lesions when considered appropriate by the interventional cardiologist. Following revascularization, patients were admitted to the Intensive Cardiovascular Care Unit (ICCU) for monitorization. This study is an extension of the biomarker sub-study of the VIP trial (NCT—NCT03606330, full name: Systemic, Pancoronary and Local Coronary Vulnerability—VIP), in which 225 patients were enrolled for the analysis of systemic vulnerability and were followed for 1 year.

#### 2.2.1. Laboratory Testing for Inflammatory Biomarkers

Blood samples were collected for all subjects within the first hour of admission in the ICCU, and were centrifugated at 3000 rotations per minute; the top layer of serum with low platelet content was collected and pipetted into sterile 10 mL vials. The serum samples were stored and refrigerated at −80 °C within 2 h from collection, until the analysis was performed. The following inflammatory serum biomarkers were tested: highly-sensitive C reactive protein (hs-CRP), interleukin 6 (IL-6), adhesion molecules (I-CAM, V-CAM, E-selectin), and matrix metalloproteinase 9 (MMP-9). The laboratory analyses were conducted in the Center for Advanced Medical and Pharmaceutical Research (CCAMF) of the University of Medicine, Pharmacy, Sciences and Technology of Târgu Mureș.

The hs-CRP levels were evaluated with the immunoturbidimetric method on the COBAD Integra 400 equipment (Roche Diagnostics, Risch-Rotkreuz, Switzerland), IL-6 with the IMMULITE 2000 XPi Immunoassay system (Siemens Healthcare GmbH, Erlangen, Germany). MMP-9 and E-selectin were evaluated with ELISA on the Dynex DSX automated system (Dynex Technologies, Chantilly, VA, USA), while the soluble levels of I-CAM and V-CAM were measured by multiplex fluorescent assay on a FLEXMAP-3D analyzer (Luminex, Austin, TX, USA).

#### 2.2.2. Evaluation of Imaging Markers

Data collection for imaging derived markers consisted of the evaluation of the degree of stenosis and the extension of coronary atherosclerosis and the left ventricular ejection fraction (LVEF). Invasive coronary angiography evaluation was performed upon admission for all the main branches of the coronary tree. Multivessel coronary artery disease was defined as the presence of >50% stenosis on all three main arteries (left anterior descending, right coronary and circumflex coronary arteries) or concomitant significant stenosis of the left main and right coronary artery. The culprit artery was identified based on the ST-segment and T wave changes on the 12-lead ECG performed in the Emergency Department. LVEF was assessed with 2D transthoracic echocardiography by using the Simpson’s biplane method, during day 5 of hospitalization with a Vivid E9 ultrasound machine (General Electrics Vingmed Ultrasound AS, Horten, Norway). All the imaging study procedures were performed by trained physicians in interventional cardiology and transthoracic echocardiography, who were blinded to the study.

#### 2.2.3. Study End-Points and Follow-Up

All included patients were followed-up for 12 months.

The primary end-point of the study was the rate of Major Adverse Cardiovascular Events (MACE), defined as a composite of all-cause mortality, reinfarction or unstable angina, hospitalization for heart failure (HF) and stroke.

The secondary end-point included the occurrence of acute phase complications during index hospitalization as a composite of:

- hemodynamic instability (cardiogenic shock, need for inotropic medication);
- new onset atrial fibrillation;
- ventricular arrhythmias (non-sustained or sustained VT not requiring electrical DC, polymorphic ventricular premature contractions);
- resuscitated cardiac arrest (out-of-hospital and in-hospital cardiac arrest);
- high-degree AV conduction abnormalities requiring temporary pacing;
- mechanical complications (rupture of free ventricular wall, interventricular septum, papillary muscle).

### 2.3. Statistical Analysis

A post-hoc statistical analysis of the collected data was performed with the use of MedCalc statistical software version 19.2.6 (MedCalc Software Ltd., Ostend, Belgium) for computing the ROC curve analysis, and GraphPad Prism version 8.4.3 (GraphPad Software, San Diego, CA, USA) for uni- and multivariable analysis. Outlier values were identified and cleared with the ROUT test, and data were tested for normality by using the D’Agostino Pearson omnibus test. Qualitative data were expressed as integer values and percentages (*n*, %), and were tested with the Chi square test and its variants when appropriate. Quantitative data were expressed as mean ± standard deviation for sets with normal distribution, as well as median for representation of non-Gaussian distributed data. The analyzed parameters were compared between groups by using the Mann Whitney or t student tests when appropriate, according to normality. The predictive capacity of biomarkers was evaluated with the receiver operating characteristics (ROC) analysis. Non-adjusted and adjusted odds ratios and 95% confidence intervals were calculated by uni- and multivariable logistic analysis of categorical parameters. The statistical significance of the study was set at an alpha of 0.05. The comparison of the analyzed parameters was performed between two groups: patients with and without the occurrence of MACE during the 1-year follow-up. In addition, a separate analysis for STEMI and NSTEMI patients was conducted by using the same group division (patients that had presented MACE versus those with no MACE during follow-up).

## 3. Results

### 3.1. Baseline Characteristics of the Study Population and End-Points

**Figure 1:**
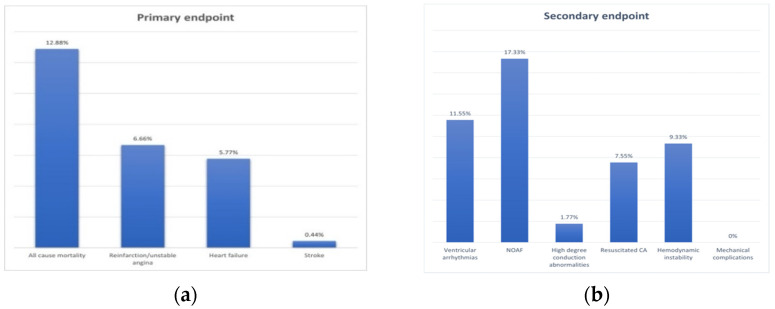
Distribution of primary (**a**) and secondary (**b**) study end-points for the overall study population.

**Figure 2:**
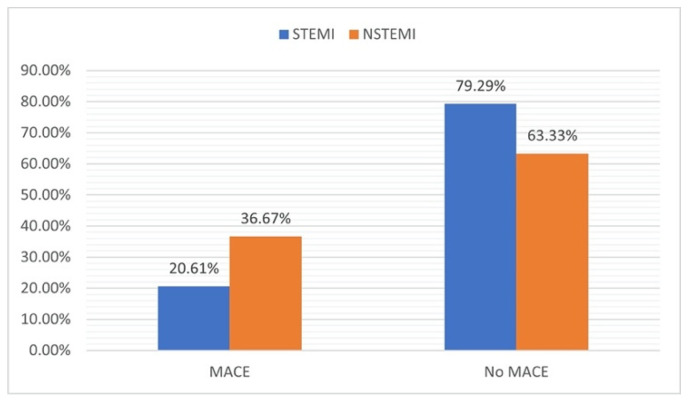
MACE rates in STEMI versus NSTEMI patients. The occurrence of 1 year MACE was significantly more frequent in patients with NSTEMI.

A total of 225 patients presenting with AMI were included in the study, with a mean age of 63.7 ± 13.4 years, and 152 (67.5%) were males. A total of 165 patients presented with STEMI (73.3%) in the first 12 h from onset of symptoms and 60 patients with NSTEMI (26.6%). The mean time from onset of symptoms to hospital admission was 12.4 ± 19.5 h for the overall study population. The most prevalent risk factor for CAD was the presence of hypertension (83.1%). The rate of composite MACE during the 1-year follow-up was 24.8% (*n* = 56). The secondary end-point was present in 30.6% (*n* = 69) of the study population, and new onset atrial fibrillation was the most frequent acute phase complication (17.3%, *n* = 17) (Figure 1). Patient demographics, index event characteristics, medical history and comorbidities, biochemical analysis and acute phase complications, as well as the comparative analysis of these characteristics between patients with and without the occurrence of MACE during follow-up, are listed in Table 1. Older age, NSTEMI diagnosis (Figure 2) and diabetes mellitus were more frequent in subjects with MACE, while smoking was less prevalent in patients that had reached the primary end-point during follow-up. There were no significant differences between groups regarding the total creatine kinase and the peak creatine kinase-MB, but the total cholesterol was lower in patients with MACE, while the glycemia measured on admission was significantly higher in decedents. None of the enrolled patients had presented mechanical complications during hospitalization.

The separate analysis of STEMI and NSTEMI patients revealed no significant differences between subjects that had presented MACE during follow up, for neither STEMI nor NSTEMI with regard to gender, general biochemical analysis, nor in respect of the occurrence of acute phase complications (Table 2). However, in the STEMI subgroup, patients that had presented the primary study end-point were significantly older (*p* < 0.001), with a lower BMI (*p* = 0.03) and with a longer time from onset of symptoms to admission (*p* = 0.01). This was not concordant with the separate analysis of NSTEMI patients (Table 2).

### 3.2. Accuracy of Serum and Imaging Markers in Predicting 1-Year MACE Rates

There were no significant differences between MACE and no-MACE subjects in respect of the levels of IL-6 and E-selectin across the overall study population and also for STEMI and NSTEMI patients. The hs-CRP levels were significantly higher in patients who developed MACE (11.1 ± 13.8 vs. 5.1 ± 4.4 mg/L, *p* = 0.03) for the total number of patients, but this was not registered when conducting a separate analysis for STEMI and NSTEMI patients. In addition, the serum levels of I-CAM (452 ± 283 vs. 220.5 ± 104.6, *p* = 0.0003) were significantly higher in patients with MACE for the overall population and STEMI, which was not consistent for NSTEMI. On the other hand, V-CAM levels were significantly higher in MACE patients for separate STEMI and NSTEMI subjects, but not for the overall population. MMP-9 was significantly elevated in patients with MACE (2255 ± 1226 vs. 1099 ± 706.1 ng/mL *p* = 0.0001) for the total number of subjects, and also during separate analyses according to the type of myocardial infarction. The results of the inflammatory serum biomarkers for the overall study population, as well as the separate analysis between the two types of MI, are illustrated in Table 3. There were no significant differences between groups regarding the presence of multivessel CAD (*p* = 0.4), nor the location of the culprit lesion in the right or left coronary tree. However, the LVEF was significantly lower in patients with MACE for all and STEMI subjects (Table 3).

To further evaluate the predictive capacity of serum and imaging markers in predicting MACE, we performed the ROC curve analysis for markers that were significantly different between the MACE and non-MACE groups, during the univariable analysis. The best cut-off values based on the Youden index and the area under de curve (AUC), with its associated sensitivity and specificity, are listed in Table 4 for the overall study population, as well as the separate analysis of STEMI and NSTEMI subjects, respectively. MMP-9 was the best predictor for the primary end-point for the overall study population, as well as during the separate analysis of STEMI and NSTEMI patients.

**Figure 3:**
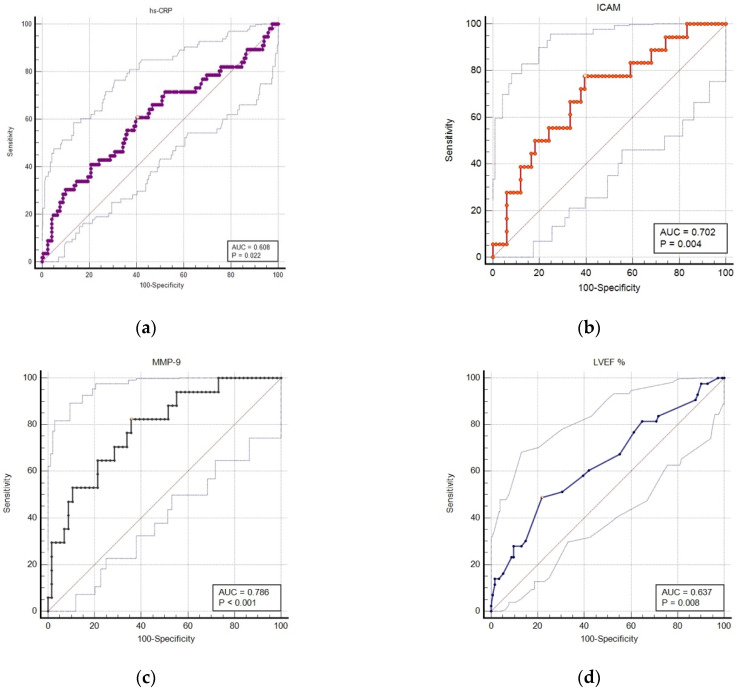
ROC analysis for the accuracy of serum and imaging markers in predicting 1 year MACE in patients with AMI. (**a**) ROC curve for hs-CRP in predicting MACE; (**b**) ROC curve for I-CAM in predicting MACE; (**c**) ROC curve for MMP-9 in predicting MACE; (**d**) ROC curve for LVEF% in predicting MACE.

Figure 3 shows the predicted probabilities for MACE during the 1-year follow-up against serum levels of hs-CRP (a), I-CAM (b), MMP-9 (c), as well as the LVEF% (d). The areas under the ROC curve (AUC) were 0.608 (*p* = 0.02), 0.702 (*p* = 0.004), 0.786 (*p* < 0.001), and 0.637 (*p* = 0.008), respectively. MMP-9 evaluated in the first day of admission for acute myocardial infarction (STEMI and NSTEMI) was the best predictor for the primary end-point. Based on the Spearman coefficient, there was a significant inverse correlation between the LVEF and hs-CRP levels (r = −0.2, 95%CI: −0.35 to −0.05, *p* = 0.007) and IL-6 (r = −0.42, 95%CI: −0.59 to −0.21, *p* = 0.0002) but not with I-CAM (*p* = 0.8), V-CAM (*p* = 0.9) and MMP-9 (*p* = 0.9) respectively. However, the correlation coefficient indicated a weak association.

### 3.3. Uni- and Multivariable Analysis for Predictors of MACE during the 1 Year Follow-Up

The univariable analysis identified the following nine statistically significant clinical, biological and morphological parameters for predicting MACE (Table 5): diabetes mellitus, less smoking, lower LVEF (<40%), decreased cholesterol levels, increased glycemia on admission, higher levels of hs-CRP, I-CAM, and MMP-9. In multivariable analysis, the most powerful predictors for MACE were serum levels of I-CAM (*p* = 0.03) and MMP-9, respectively, after adjustments were performed (Table 5). The predictive accuracy of the proposed model, to include DM, smoking, total cholesterol, glycemia on admission, LVEF < 40%, the composite of acute phase complications, hsCRP, I-CAM and MMP-9, was characterized by the following statistical parameters: AUC: 0.773 (95%CI: 0.68–0.86), standard error: 0.04, *p* < 0.0001, negative predictive power: 80.5%, positive predictive power: 73.0%.

## 4. Discussions

The present study aimed to demonstrate the predictive capacity of serum biomarkers illustrating systemic inflammation, in relation to morphological characteristics indicating the anatomy of the CAD and left ventricular function, in patients with revascularized AMI, during a 1-year follow-up period. The main findings of our study were that patients that had presented MACE during the 1-year follow-up presented significantly higher levels of serum inflammatory biomarkers sampled at 1 h from hospital admission (hs-CRP, MMP-9, I-CAM), for the overall study population. In the case of STEMI patients alone, hs-CRP was not different between MACE groups, but I-CAM, V-CAM and MMP-9 were significantly higher in those that had presented the primary study end-point during follow-up. For NSTEMI, only V-CAM was significantly higher for MACE patients. The best predictors for 1-year adverse events included elevated levels of I-CAM > 239.7 ng/L and an MMP-9 level of over 1155 ng/mL, respectively, after adjustments for diabetes, hs-CRP, acute phase complications and LVEF for the overall study population. For STEMI patients, the best predictors for the primary study end-point included I-CAM > 239.7 ng/mL, and MMP-9 > 1393 ng/mL, while for NSTEMI subjects, none of the analyzed serum biomarkers had reached significant predictive capacity. Surprisingly, there was no significant association between in-hospital adverse events and any of the analyzed serum biomarkers, nor the angiographical characteristics of LV systolic function of the study population. Although the serum levels of hs-CRP, MMP-9 and I-CAM were more elevated in patients that had presented acute phase complications (electrical or hemodynamic), the difference did not reach statistical significance neither for the total number of patients, nor during separate analysis for STEMI and NSTEMI.

The study results support prior evidence indicating that an enhanced inflammatory response following an ACS can alter patient outcomes. Initial inflammation following acute myocardial injury is responsible for wound healing, but an excessive immune response leads to excessive left ventricular remodeling, HF, vulnerabilization of non-culprit lesions with subsequent coronary events, and it may also trigger electrical vulnerability of the myocardium, with the associated arrhythmias and sudden death [36,37]. Nevertheless, the present study offers new data regarding the integration of biological and morphological parameters into a prediction model for MACE. The analysis included a panel of inflammatory serum biomarkers (acute phase reactants, adhesion molecules and matrix metalloproteinases), as well as several clinical and imaging parameters for the identification of the best prediction model for adverse events at 1 year after the acute event. To conduct a precise analysis of the biological inflammatory biomarkers in predicting outcomes following AMI, patients that had presented associated conditions linked to an enhanced inflammatory response were excluded from the study.

Additional findings of our study included a higher rate of smokers in the non-MACE group, thus confirming the “smokers paradox” yet again. This was consistent for the overall study population, but also for NSTEMI and STEMI subjects. Several previous studies have found that, while presenting a higher risk for developing ACS, smokers paradoxically present better outcomes and less complications during hospitalization, compared to non-smokers. However, the improved survival of these patients has not been consistent in the PCI era, during which there have been higher complications rates, including in-stent restenosis and repeated revascularization procedures [38,39,40].

Intravascular and extravascular inflammation is triggered and quantified by local and systemic cytokines or other players in the inflammatory retort, including cell adhesion molecules and matrix metalloproteinases [41]. All these biomarkers have been analyzed from various angles with respect to inflammatory activation related to ACS [42]. Acute myocardial injury and ischemia triggers an inflammatory response, which influences infarct size, myocardial fibrosis and remodeling, as well as progression towards HF and other complications related to AMI [43]. Various inflammatory biomarkers have been studied in relation to ACSs, including acute phase reactants, cytokines or cell adhesion molecules [20,21,27].

CRP evaluated with highly sensitive assays has been considered as the prototype serum inflammatory biomarker, with a proven strong predictive capacity in a wide array of cardiovascular disorders [44,45,46]. The prognostic utility of hs-CRP levels in AMI has been reported by several authors, including in-hospital adverse events or at 6 months [47,48]. In our study, hs-CRP levels were significantly higher in acute myocardial infarction patients that had presented the primary end-point but not during separate analysis according to the type of AMI. Nevertheless, all hs-CRP values were higher in patients that had presented 1-year adverse events. This suggests that an excessive inflammatory response during the acute phase of AMI leads to worse outcomes, regardless of the type of myocardial infarction. Contrarily, no significant relationship between hs-CRP levels and acute phase complications was found in our study, which could be due to the exclusion of patients that had deceased during hospitalization.

IL-6 has been associated with vascular inflammation, plaque vulnerability, and worse outcomes in ACS patients. A study of 4939 patients with MI showed that increased IL-6 levels were linked to a higher risk of cardiovascular death and HF [19]. Ammirati et al. have shown that AMI patients with simultaneously increased levels of IL-6 and Il-10 were more prone to have impaired LV systolic dysfunction upon discharge, and also an increased risk of death during a 6-month follow-up period [49]. However, in our study, there was no difference in IL-6 levels between patients that had and had not reached the primary end-point, for all patients and also during separate analysis according to the type of AMI.

Soluble plasma levels of cell adhesion molecules may provide useful information on risk stratification and disease severity [50,51,52]. Serum levels of several cellular adhesion molecules have been shown to increase in patients with significant CAD, and more so in ACS [53,54]. V-CAM and I-CAM have been shown to predict MACE during hospitalization or during various follow-up periods [25,51,55]. A recent study on STEMI patients found that I-CAM and V-CAM were the best predictors for in-hospital development of HF [20]. V-CAM has been shown to provide a powerful predictive value for MACE, including new ACS, hospitalization for chest pain, and cardiovascular death in patients with ACS [25]. A substudy derived from the PRIME cohort found that elevated serum levels of I-CAM, but not V-CAM, were associated with an increased risk of AMI, cardiovascular mortality and angina during a 5-year follow-up [56]. Similarly, in our study, all patients with MACE showed significantly higher plasma levels of I-CAM, but not V-CAM or E-selectin, thus suggesting that, in this context, the evaluated adhesion molecules act differently in the inflammatory reaction following an ACS. The difference is emphasized also during the STEMI and NSTEMI separate analysis, which revealed that, in the case of STEMI, I-CAM and V-CAM were significantly higher in MACE patients, whereas in NSTEMI, only V-CAM was higher. In addition, the cut-off value of 239.7 ng/mL for I-CAM was a significant predictor for 1-year adverse events, but not for in-hospital complications for all patients. Furthermore, I-CAM also predicted MACE rates in STEMI patients but not NSTEMI. V-CAM, on the other hand, did not show significant predictive power in the case of all patients nor in NSTEMI, but a cut-off value of >877.9 ng/mL significantly predicted 1-year adverse events. This leads to the assumption that the soluble form of I-CAM is a marker for long-term patient outcome in the case of AMI patients, but V-CAM may be used as a significant predictor for adverse events solely in STEMI subjects. The statistical power of I-CAM was also found during multiple logistic regression analysis, which further indicates its use for patient prognosis and myocardial healing.

MMPs play a central role in the process of plaque rupture and myocardial tissue remodeling after an acute ischemic event, by degrading the extracellular matrix, mainly type IV collagen and elastin, and enabling the migration of inflammatory mediators across tissues [57]. Serum levels of MMP-9 increase during an acute coronary event and have also been linked to patient outcomes [58]. In a study of 155 AMI patients undergoing primary PCI, MMP-9 levels above the cut-off value established at 398.2 ng/mL, was an independent predictor for in-hospital mortality [59]. In addition, the persistently elevated levels of MMP-9 during the recovery phase from AMI has been associated with a poor cardiovascular outcome across a follow-up period of 6 years after the acute event [21]. In our study, MMP-9 was the best predictor for 1-year MACE, even after adjustment for LVEF and comorbidities, and also cholesterol levels and hs-CRP, for all AMI patients, and more specifically in STEMI. However, MMP-9 was not associated with the complications occurring during hospitalization for the acute event neither during the global nor separate analysis.

### 4.1. Imaging Predictors for MACE in the Context of an Enhanced Systemic Inflammation

The present study did not find any significant association between the presence of multivessel CAD and 1-year MACE rates, nor the location of the culprit lesion within the coronary tree. However, several previous studies have shown that multivessel CAD is a significant determinant of adverse events [29,60,61]. Although the morphological parameters characterizing the coronary anatomy did not differ between our study groups, there was a significant association between impaired LVEF, examined at day 5 from the index event, and the occurrence of MACE, for all patients and STEMI, but not for NSTEMI subjects. Impaired LVEF has been extensively proven to be one of the most important prognostic markers for short and long-term patient outcomes in various clinical settings. In the context of the present study results, it is not clear whether a decreased LVEF had led to an enhanced inflammatory response, or if the increased levels of inflammatory molecules had contributed to the impaired left ventricular function. Based on the Spearman coefficient, we observed that the LVEF was inversely correlated with the hs-CRP and IL-6 levels, but not with the other analyzed serum biomarkers. However, the correlation coefficient indicated a weak association. Several studies have shown that increase levels of hs-CRP, IL-6 and other biological markers promote the LV remodeling process following an acute coronary event [62,63,64]. A study of 369 patients with AMI found a significant association between hospital admission levels of IL-6 and a larger infarct size and decreased LV function, evaluated via cardiac magnetic resonance and echocardiography [65]. Peak hs-CRP levels during the acute phase following a myocardial infarction (median of 12.10 mg/L) has been linked to LV dysfunction at 1 month after the acute event, but also with the serum levels of highly sensitive troponin T and white blood cell count [66].

The most powerful risk factors for MACE, during the analysis of all included patients (STEMI and NSTEMI), identified with univariable analysis (diabetes, depressed LVEF, increased levels of hs-CRP, I-CAM and MMP-9), were included in a multivariable model for predicting MACE. The proposed model proved an increased predictive capacity with an AUC of 0.773, and a negative predictive power of 80.5%. Parameters included in the proposed model could be included into established risk prediction tools that encompass clinical, laboratory and imaging parameters, in order to achieve superior prognostic value.

The biological status of AMI patients, characterized by an enhanced inflammatory state, leads to the alteration of morphological characteristics including the extension of coronary atherosclerosis, the vulnerabilization of non-culprit plaques and the impairment of the left ventricular function, thus influencing patient outcomes. The systemic vulnerability of patients that have suffered an acute coronary event is still a hot topic in cardiovascular research, as several predictive markers are still arising. The concept of a cardiovascular vulnerable patient refers to systemic vulnerability including systemic inflammation and increased thrombogenicity, and also local vulnerability, that encompasses unstable coronary plaques and vulnerable myocardium [67]. Both systemic and local coronary vulnerability should be included in models that can predict patient outcomes, both in the short and long term, following AMI. Nevertheless, the most important result of the present study was that inflammatory serum biomarkers (I-CAM and MMP-9) were better predictors for 1 year MACE, in comparison to the left ventricular systolic function quantified by the LVEF or the severity of CAD.

### 4.2. Clinical Applications

In addition to providing valuable prognostic insights, the evaluation of several inflammatory biomarkers in a panel approach can also provide therapeutic targets for improving patient outcomes. Several completed or ongoing clinical trials have tested the effects of anti-inflammatory therapies in AMI patients [68]. The Canakinumab Anti-inflammatory Thrombosis Outcome Study (CANTOS) trial showed that patients with a history of AMI and an enhanced inflammatory response illustrated by hs-CRP levels of >2 mg/L, who were treated with 150 mg of canakinumab (monoclonal antibody targeting Il-1β), presented significantly lower MACE rates during a median follow-up of 3.7 years, as well as lower hs-CRP levels during follow-up, in comparison with the placebo group, and also with the lower dose canakinumab group (50 mg/day) [69]. Our study advocates for the stratification of patients at risk of adverse events, based on the serum levels of three inflammatory biomarkers (hs-CRP, I-CAM and MMP-9). This stratification may potentially improve prognostic accuracy, modulate patient follow-up, and identify subjects that may be suitable for targeted novel anti-inflammatory therapies [70].

### 4.3. Study Limitations and Future Perspectives

Although the present study offers additional information on the prognostic role of the biological and morphological characteristics of patients with AMI, it has several limitations. Firstly, the primary end-point included all-cause mortality, but the evaluation of cardiovascular-disease related deaths would add important information. The study also lacks data regarding staged revascularization of lesions arising from non-infarct related arteries. This was a single center study of consecutive patients presenting with acute myocardial infarction with a small sample size, excluding patients with unstable angina or chronic coronary syndromes. A further analysis of a larger study population, which would also include patients with unstable angina and stable CAD, is needed to validate the initial results of the current analysis. For future perspectives, in addition to a longer follow-up period, it would be of great interest to analyze the impact of dynamic serial measurements of the analyzed serum biomarkers, as well as the persistence of inflammation beyond the acute phase, in modifying the prognosis of these patients. The evaluation of the effect of a dual antiplatelet therapy regimen as well as a statin treatment of the inflammatory response and its relationship to 1 year patient outcomes may add additional value to the current results and will be included in a future analysis.

## 5. Conclusions

Patients with increased levels of hs-CRP, I-CAM and MMP-9 sampled at one hour following an AMI, and lower LVEF, present a significantly higher risk of developing major adverse cardio- and cerebrovascular events at 1 year. The most powerful predictors for MACE in this setting were an I-CAM level of >239.7 ng/mL and an MMP-9 of over 1155 ng/mL, even after adjustments for clinical and other biological and morphological markers, in patients with AMI. For STEMI, the most powerful predictors for MACE included I-CAM > 239.7 ng/mL, V-CAM > 877.9 ng/mL and MMP-9 > 1393 ng/mL. The most important finding was that inflammatory biomarkers assayed during the acute phase of the coronary event were more powerful predictors for MACE than the LVEF. Our study advocates for the stratification of patients with acute myocardial infarction at risk of developing adverse events, based on the serum levels of two inflammatory biomarkers (I-CAM and MMP-9), regardless of presenting with STEMI or NSTEMI. This stratification may potentially improve prognostic accuracy, modulate patient follow-up, and identify subjects at risk for long term adverse events. Parameters included in the proposed model could be included into established risk prediction tools that encompass clinical, laboratory and imaging parameters, in order to achieve superior prognostic value.

## Figures and Tables

**Table 1:** Clinical and biochemical characteristics of the study population and comparative analysis between the two study groups.

Variable	Total *n* = 225	Primary End-Point Reached during Follow-Up		*p* Value
		Yes *n* = 56	No *n* = 169	
**Patient demographics**				
**Age, y, mean ± SD (median)**	63.7 ± 13.4 (65)	70 ± 10.5 (71)	61.7 ± 13.2 (63)	0.0003
**Gender, male *n* (%)**	152 (67.5%)	36 (64.2%)	116 (68.6%)	0.5
**BMI kg/m^2^**	28 ± 5.4	27.3 ± 5	28.3 ± 5.6	0.1
**Index event characteristics**				
**STEMI *n* (%)**	165 (73.3%)	34 (20.6%)	131 (79.3%)	0.01
**NSTEMI *n* (%)**	60 (26.6%)	22 (36.6%)	38 (63.3%)	
**Time from onset of symptoms to admission, hrs, mean ± SD (median) for total**	12.4 ± 19.5 (8)	19.6 ± 33.6 (10)	9.8 ± 9.7 (7)	0.3
**Medical history and comorbidities**				
**HTN *n* (%)**	187 (83.1%)	50 (89.2%)	137 (81%)	0.2
**DM *n* (%)**	60 (26.6%)	21 (37.5%)	39 (23.1%)	0.03
**Smoking *n* (%)**	90 (40%)	12 (21.4%)	78 (46.1%)	0.001
**Dyslipidemia *n* (%)**	69 (17.2%)	16 (28.5%)	53 (31.3%)	0.6
**Stroke *n* (%)**	19 (8.4%)	7 (12.5%)	12 (7.1%)	0.3
**Previous MI *n* (%)**	20 (8.8%)	8 (14.2%)	12 (7.1%)	0.1
**PAD *n* (%)**	11 (4.8%)	4 (7.1%)	7 (4.1%)	0.4
**Obesity *n* (%)**	45 (20%)	12 (21.4%)	33 (14.6%)	0.7
**Biochemical analysis for renal, metabolic and myocardial necrosis markers, mean ± SD (median)**				
**Peak CK MB (U/L)**	80.9 ± 172.2 (21.9)	79.8 ± 130.6 (24.2)	133.6 ± 59.2 (21.9)	0.6
**Creatine kinase (U/L)**	1480 ± 1631 (868)	1367 ± 1432 (867)	1516 ± 1692 (868)	0.7
**Total cholesterol (mg/dL)**	187.8 ± 50.5 (186.5)	175.7 ± 48.7 (175)	192 ± 50.5 (190.2)	0.03
**Triglycerides (mg/dL)**	168.2 ± 112.5 (139.2)	167.9 ± 140.5 (124)	168.3 ± 101.1 (149.5)	0.1
**Glycemia on admission (mg/dL)**	142.6 ± 61.9 (121.5)	155 ± 67.4 (139)	138.3 ± 59.6 (117)	0.01
**eGFR (mL/min)**	95.1 ± 37.4 (95.5)	87.2 ± 39.7 (92.5)	97.7 ± 36 (97.4)	0.1
**Acute phase complications**				
**Ventricular arrhythmias *n* (%)**	26 (11.5%)	8 (14.2%)	18 (10.6%)	0.6
**NOAF *n* (%)**	39 (17.3%)	13 (23.2%)	26 (15.3%)	0.1
**High degree AV conduction abnormalities *n* (%)**	4 (1.7%)	1 (1.7%)	3 (1.7%)	0.9
**Resuscitated CA *n* (%)**	17 (7.5%)	6 (10.7%)	11 (6.5%)	0.3
**Hemodynamic instability *n* (%)**	21 (9.3%)	7 (12.5%)	14 (8.2%)	0.4
**Mechanical complications *n* (%)**	0	0	0	n.a.
**Composite of all acute complications *n* (%)**	69 (30.6%)	21 (37.5%)	48 (28.4%)	0.2

eGFR—estimated glomerular filtration rate, NOAF—new onset atrial fibrillation, CA–cardiac arrest.

**Table 2:** Separate analysis of STEMI and NSTEMI populations in regard to clinical and biochemical characteristics and occurrence of MACE.

	STEMI Patients				NSTEMI Patients			
Variable	Total *n* = 165 (73.3%)	Primary End-Point Reached during Follow-Up		*p* Value	Total *n* = 60 (26.6%)	Primary End-Point Reached during Follow-Up		*p* Value
		Yes *n* = 34 (20.6%)	No *n* = 131 (79.3%)			Yes *n* = 22 (36.6%)	No *n* = 38 (63.3%)	
**Age, y, mean ± SD (median)**	61.4 ± 14.0 (62)	69 ± 14 (70)	59.7 ± 13.4 (60)	<0.001	69.6 ± 9.4 (71)	71.1 ± 9.3 (74)	68.8 ± 9.5 (69)	0.3
**Gender, male *n* (%)**	123	23 (67.5%)	100 (76.3%)	0.3	29	13 (59%)	16 (42.1%)	0.3
**BMI kg/m^2^**	28.1 ± 5.5 (26.9)	26.5 ± 4.3 (26.2)	28.4 ± 5.8 (27.1)	0.03	28 ± 5.1 (27.4)	28.5 ± 5.8 (27.7)	27.7 ± 4.8 (27.2)	0.5
**Time from onset of symptoms to admission, hrs, mean ± SD (median)**	7.3 ± 3.1 (7)	9.2 ± 0.2.4 (10)	6.6 ± 3.1 (6)	0.01	23.5 ± 31.1 (10)	38.6 ± 54.2 (4.5)	17 ± 14.9 (12)	0.4
**Medical history and comorbidities**								
**HTN *n* (%)**	132 (80%)	30 (88.2%)	102 (77.2%)	0.2	55 (91.6%)	20 (90.9%)	35 (92.1%)	0.9
**DM *n* (%)**	34 (20.6%)	7 (20%)	28 (21.2%)	0.9	25 (41.6%)	14 (63.6%)	11 (28.9%)	0.01
**Smoking *n* (%)**	78 (29%)	11 (32.5%)	67 (51.1%)	0.05	12 (20%)	1 (4.55%)	11 (81.8%)	0.001
**Dyslipidemia *n* (%)**	52 (31.5%)	10 (29.4%)	42 (32%)	0.7	17 (28.3%)	6 (27.2%)	11 (28.9%)	0.8
**Stroke *n* (%)**	9 (5.4%)	3 (8.8%)	6 (4.5%)	0.3	10 (15.3%)	4 (18.1%)	6 (15.7%)	0.9
**Previous MI *n* (%)**	5 (3%)	1 (2.9%)	4 (3.0%)	0.9	15 (16.6%)	7 (31.8%)	8 (21.0%)	0.5
**PAD *n* (%)**	4 (2.4%)	0 (0%)	4 (3.0%)	0.5	7 (11.6%)	4 (18.1%)	3 (7.8%)	0.4
**Obesity *n* (%)**	24 (14.5%)	3 (8.8%)	21 (16%)	0.4	21 (35%)	9 (40.9%)	12 (31.5%)	0.6
**Biochemical analysis for renal, metabolic and myocardial necrosis markers, mean ± SD (median)**								
**Peak CK MB (U/L)**	103.8 ± 205.5 (22.0)	105.9 ± 164.2 (24.05)	101.9 ± 245.8 (21.95)	0.9	32.8 ± 35.8 (19.3)	40.6 ± 39.4 (28.6)	44 ± 38.5 (44)	0.2
**Creatine kinase (U/L)**	1781 ± 1756 (1233)	1751 ± 1613 (1232)	1789 ± 1796 (1233)	0.9	668.3 ± 795.9 (320)	800.2 ± 871.1 (580)	593.4 ± 752.1 (260)	0.2
**Total cholesterol (mg/dL)**	188.7 ± 51.6 (188)	178.3 ± 50.2 (175)	191.6 ± 51.8 (189.1)	0.1	185.1 ± 47.57	171.8 ± 47.3	193.3 ± 46.4	0.1
**Triglycerides (mg/dL)**	167.7 ± 114 (134)	162.1 ± 144.4 (112.7)	169.2 ± 104.6 (147)	0.1	169.6 ± 109.2	176.8 ± 137.4	165.4 ± 90.2	0.4
**Glycemia on admission (mg/dL)**	140.5 ± 56.2 (120)	142.4 ± 53.5 (130)	140 ± 57.2 (119)	0.3	147.9 ± 75	173.8 ± 82	132.9 ± 67.3	0.02
**eGFR (mL/min)**	95 ± 35.8 (95.3)	89.4 ± 38.7 (95.3)	96.5 ± 35.1 (95.2)	0.3	95.2 ± 40.7	83.6 ± 42.3	101.1 ± 39.2	0.2
**Acute phase complications *n* (%)**								
**Ventricular arrhythmias**	20 (12.1%)	5 (14.7%)	15 (11.4%)	0.5	7 (11.6%)	3 (13.6%)	4 (10.5%)	0.6
**NOAF**	27 (16.3%)	8 (23.5%)	19 (14.5%)	0.3	12 (20%)	5 (22.7%)	7 (18.4%)	0.7
**High degree AV conduction abnormalities**	3 (1.8%)	0 (0%)	3 (2.2%)	0.9	1 (1.6%)	1 (4.5%)	0 (0%)	0.3
**Resuscitated CA**	17(10.3%)	4 (12.1%)	13 (7.6%)	0.4	3 (5%)	2 (9.0%)	1 (2.5%)	0.2
**Hemodynamic instability**	14 (8.48%)	4 (11.7%)	10 (9.9%)	0.7	5 (8.3%)	3 (13.6%)	2 (5.26%)	0.3
**Mechanical complications**	0	0	0	na	0	0	0	na
**Composite of all acute complications**	52 (31.5%)	13 (38.2%)	39 (29.7%)	0.3	17 (28.3%)	8 (36.3%)	9 (23.6%)	0.4

**Table 3:** Serum biomarkers and imaging parameters.

	Total Study Population (STEMI + NSTEMI)				STEMI Patients				NSTEMI Patients			
Variable	Total *n* = 225	Primary End-Point Reached during Follow-Up		*p* Value	Total *n* = 165 (73.3%)	Primary End-Point Reached during Follow-Up		*p* Value	Total *n* = 60 (26.6%)	Primary End-Point Reached during Follow-Up		*p* Value
		Yes *n* = 56	No *n* = 169			Yes *n* = 34 (20.6%)	No *n* = 131 (79.3%)			Yes *n* = 22 (36.6%)	No *n* = 38 (63.3%)	
**Serum inflammatory biomarkers, mean ± SD (median)**												
**Hs-CRP (mg/L)**	5.2 ± 4.5 (3.6)	11.1 ± 13.8 (5.7)	5.1 ± 4.4 (3.4)	0.03	4.6 ± 3.9 (3.4)	5.6 ± 5.7 (4.3)	4.2 ± 3.3 (3.3)	0.6	6.6 ± 5.2 (5.5)	11.24 ± 11.8 (6.7)	6.8 ± 5.7 (4.7)	0.08
**Il-6 (pg/mL)**	8.0 ± 5.5 (6.8)	8.9 ± 7.0 (6.9)	8.7 ± 6.5 (7.0)	0.9	20 ± 44.9 (8.4)	34.6 ± 94.6 (8.6)	16.8 ± 23.3 (8.4)	0.6	16.0 ± 30.6 (7.4)	12.9 ± 17.1 (5.4)	17.6 ± 35.8 (7.8)	0.8
**I-CAM (ng/mL)**	250.0 ± 133.9 (215.4)	452 ± 283 (390.8)	220.5 ± 104.6 (201.1)	0.0003	342 ± 237.4 (246.4)	490.1 ± 226.2 (239.4)	309.1 ± 226.2 (227.6)	0.006	248.4 ± 226.1 (179.5)	375.7 ± 351.6 (214.8)	184.7 ± 62.5 (171.7)	0.1
**V-CAM (ng/mL)**	966.5 ± 248.3 (895.2)	1045 ± 317.7 (895.2)	953.3 ± 235.0 (901.5)	0.4	1002 ± 339.5 (895.2)	1274 ± 569.2 (1067)	938.4 ± 224.9 (894.6)	0.02	845.4 ± 245.8 (927)	767.2 ± 69.7 (757.8)	994 ± 255.2 (948.6)	0.01
**E-selectin (ng/mL)**	71.7 ± 30.1 (67.8)	74.7 ± 28 (72.7)	70.2 ± 30.8 (64.7)	0.3	72.4 ± 29.8 (68.8)	78.2 ± 23.3 (73.2)	71.2 (32.2)	0.2	63.9 ± 33.9 (57.6)	61 ± 40.6 (49.9)	66 ± 30.1 (58)	0.7
**MMP-9 (ng/mL)**	1285 ± 843.7 (1117)	2255 ± 1226 (1937)	1099 ± 706.1 (1020)	0.0001	1412 ± 1067 (1135)	2554 ± 1275 (2249)	1173 ± 856.9 (1101)	0.001	1452 ± 966 (1110)	1919 ± 1155 (1608)	1096 ± 683 (846)	0.09
**Imaging markers**												
**LVEF%** **(Simpson’s biplane)**	44.2 ± 6.5 (45)	41.4 ± 7.6 (42)	45.1 ± 6.1 (45)	0.005	44 ± 6.3 (45)	40.8 ± 7.3 (43)	44.8 ± 5.7 (45)	0.01	44.4 ± 8.1 (45)	42.2 ± 8.1 (40)	46.3 ± 7.8 (45.5)	0.1
Multivessel CAD *n* (%) *	123 (54.6%)	33 (58.9%)	90 (53.2%)	0.4	76 (46%)	15 (44.1%)	64 (48.8%)	0.6	44	18 (81.8%)	26 (68.4%)	0.3
**Left coronary artery culprit *n* (%)**	158 (70.2%)	41 (73.2%)	117 (69.2%)	0.5	108 (65.4%)	24 (70.5%)	84	0.4	50 (83.3%)	17 (77.2%)	33 (86.8%)	0.4
**Right coronary artery culprit *n* (%)**	67 (29.7%)	15 (26.7%)	52 (30.7%)	0.5	57 (34.5%)	10 (29.4%)	47	0.4	10 (16.6%)	5 (22.7%)	5 (13.1%)	0.4

* Multivessel CAD—defined as the presence of >50% stenosis on all three main arteries (left anterior descending, right coronary and circumflex coronary arteries) or concomitant significant stenosis of the left main and right coronary artery.

**Table 4:** ROC curve analysis for serum and imaging markers in predicting MACE.

Total Study Population (STEMI + NSTEMI)								
Parameter	AUC	95%CI for AUC	*z* Statistic	Youden Index	Cut off Value for Predicting MACE	Sensitivity %	Specificity %	*p* Value
hs-CRP (mg/L)	0.608	0.54–0.67	2.29	0.204	>5.6	60.7	59.7	0.02
I-CAM (ng/mL)	0.702	0.59–0.79	2.90	0.383	>239.7	77.7	60.6	0.004
V-CAM (ng/mL)	0.600	0.48–0.70	1.29	0.20	>975.4	61.1	59.1	0.6
MMP 9 (ng/mL)	0.786	0.67–0.87	4.61	0.466	>1155	82.3	64.2	<0.001
LVEF%	0.637	0.55–0.71	2.64	0.269	≤40	48.8	78.0	0.008
**STEMI patients**								
hs-CRP (mg/L)	0.572	0.49–0.64	1.18	0.191	>13.0	34.38	0.78	0.2
I-CAM (ng/mL)	0.747	0.62–0.84	3.52	0.472	>239.7	91.67	1.85	<0.001
V-CAM (ng/mL)	0.715	0.58–0.82	2.45	0.355	>877.9	90.91	2.13	0.01
MMP-9 (ng/mL)	0.828	0.70–0.91	4.49	0.596	>1393	88.89	2.27	<0.001
LVEF%	0.652	0.56–0.73	2.48	0.25	≤40	46.71	100.0	0.01
**NSTEMI patients**								
hs-CRP (mg/L)	0.633	0.49–0.75	1.69	0.27	>5.7	77.2	2.6	0.09
I-CAM (ng/mL)	0.667	0.41–0.86	1.08	0.33	>234.0	50	8.3	0.2
V-CAM (ng/mL)	0.528	0.28–0.76	0.18	0.25	≤852.1	50.0	91.6	0.8
MMP 9 (ng/mL)	0.729	0.48–0.90	1.85	0.45	>849	87.5	8.33	0.06
LVEF%	0.640	0.46–0.79	1.5	2.44	≤37	29.4	95.0	0.1

**Table 5:** Uni- and multivariable logistic analysis for predictors of MACE during the 1 year follow-up.

**Univariable Analysis**			
**Variable**	**OR**	**95% CI for OR**	***p***
Gender	0.8	0.44–1.51	0.5
HTN	1.9	0.78–4.70	0.2
DM	2.0	1.06–3.81	0.03
Smoking	0.3	0.16–0.63	0.001
Dyslipidemia	0.8	0.46–1.67	0.6
Stroke	1.8	0.72–4.89	0.3
Previous MI	2.1	0.84–5.46	0.1
PAD	1.7	0.56–5.89	0.4
Obesity	1.1	0.53–2.32	0.7
Multivessel CAD	1.2	0.67–2.23	0.4
Total cholesterol	0.9	0.98–0.99	0.04
Glycemia on admission	1.0	0.99–1.00	0.09
LVEF < 40%	2.7	1.07–5.67	0.03
hs-CRP (mg/L)	2.3	1.22–4.30	0.007
ICAM (ng/mL)	5.0	1.62–15.03	0.007
MMP-9 (ng/mL)	8.4	2.22–29.23	0.0009
**Multivariable Analysis**			
**Variable**	**Adjusted OR**	**95% CI for adjusted OR**	***p***
DM	4.4	0.31–80.14	0.2
Smoking	0.4	0.03–4.06	0.5
Total cholesterol	0.9	0.98–1.00	0.2
LVEF	0.9	0.83–1.04	0.2
Acute phase complications *	1.1	0.42–2.92	0.7
hs-CRP (mg/L)	1.5	0.62–0.97	0.3
ICAM (ng/mL)	3.2	1.11–9.88	0.03
MMP-9 (ng/mL)	3.6	1.21–11.49	0.02

* Acute phase complications include: hemodynamic instability (cardiogenic shock, need for inotropic medication), new onset atrial fibrillation, ventricular arrhythmias, resuscitated cardiac arrest, high degree AV conduction abnormalities requiring temporary pacing, mechanical complications.

## Data Availability

The data presented in this study are available on request from the corresponding authors. The data are not publicly available due to privacy and ethical restrictions (containing information that could compromise the privacy of the study subjects).
